# Neural Wiskott-Aldrich syndrome protein (N-WASP) promotes distant metastasis in pancreatic ductal adenocarcinoma via activation of LOXL2

**DOI:** 10.32604/or.2024.044029

**Published:** 2024-03-20

**Authors:** HYUNG SUN KIM, YUN SUN LEE, SEUNG MYUNG DONG, HYO JUNG KIM, DA EUN LEE, HYEON WOONG KANG, MYEONG JIN KIM, JOON SEONG PARK

**Affiliations:** 1 Department of Surgery, Gangnam Severance Hospital, Yonsei University College of Medicine, Seoul, Korea; 2 Department of Surgery, Graduate School of Medical Science, Brain Korea 21 Project, Yonsei University College of Medicine, Seoul, Korea; 3 Gibiome Co., Ltd., Gyeonggi-do, Korea

**Keywords:** Pancreatic cancer, Neural Wiskott-Aldrich syndrome protein (N-WASP) signaling, Metastasis, Epithelial–mesenchymal transition (EMT), Lysyl oxidase-like 2 (LOXL2)

## Abstract

Pancreatic ductal adenocarcinoma (PDAC) is one of the most aggressive solid malignancies. A specific mechanism of its metastasis has not been established. In this study, we investigated whether Neural Wiskott-Aldrich syndrome protein (N-WASP) plays a role in distant metastasis of PDAC. We found that N-WASP is markedly expressed in clinical patients with PDAC. Clinical analysis showed a notably more distant metastatic pattern in the N-WASP-high group compared to the N-WASP-low group. N-WASP was noted to be a novel mediator of epithelial-mesenchymal transition (EMT) via gene expression profile studies. Knockdown of N-WASP in pancreatic cancer cells significantly inhibited cell invasion, migration, and EMT. We also observed positive association of lysyl oxidase-like 2 (LOXL2) and focal adhesion kinase (FAK) with the N-WASP-mediated response, wherein EMT and invadopodia function were modulated. Both N-WASP and LOXL2 depletion significantly reduced the incidence of liver and lung metastatic lesions in orthotopic mouse models of pancreatic cancer. These results elucidate a novel role for N-WASP signaling associated with LOXL2 in EMT and invadopodia function, with respect to regulation of intercellular communication in tumor cells for promoting pancreatic cancer metastasis. These findings may aid in the development of therapeutic strategies against pancreatic cancer.

## Introduction

Pancreatic ductal adenocarcinoma (PDAC) is known to be a fatal disease, with an overall 5-year survival rate remaining unchanged, despite advancements in early screening and diagnosis. Even after surgery, early recurrence rate, local recurrence, and distant metastasis tend to be high in pancreatic cancer. Tumor metastasis is a multistep process, wherein tumor cells disseminate from their primary site and form secondary tumors at a distant site. Metastasis occurs through a series of steps, such as local invasion, intravasation, transport, extravasation, and colonization. Epithelial–mesenchymal transition (EMT) is crucial a developmental program for promoting metastasis in epithelium-derived carcinoma [1–4].

EMT transcription factors can also induce the formation of specialized subcellular structures called “invadopodia,” which invade the local extracellular matrix (ECM). Invadopodia play a key role in metastasis by local invasion [1,5–8]. EMT not only allows carcinoma cells to dissociate from each other but also provides the ability to degrade ECM for single-cell invasion, thereby helping initiate the metastatic cascade. Critical initial steps in pancreatic cancer metastasis involve the detachment and invasion of tumor cells into surrounding tissues, which require changes to the adhesive and migratory properties of tumor cells. It can be achieved partly through cell polarization and the extension of actin-rich membrane structures in the direction of movements, such as filopodia, lamellipodia, and invadopodia occurring in invasive cancer cells [7,9–12]. Recent studies suggest that focal adhesion kinase (FAK) is involved in cancer progression through signaling via integrins and other cell surface receptors and plays an important role in the regulation of cell migration. Membrane protrusions formation, which is crucial for cell motility, is controlled by the rearrangement of the actin cytoskeleton. FAK, which is crucial for FA turnover, inhibits ECM proteolysis. The role of FAK in cancer cells is regulation of invadopodia formation and function [13–16].

Neural Wiskott-Aldrich syndrome protein (N-WASP) is a ubiquitously expressed member of the WASP family. This protein family is involved in changing cell morphology (e.g., invadopodium formation) and growth and exhibits a correlation with certain cancer phenotypes. A high level of N-WASP expression positively correlates with progression and/or poor outcome in lung, pancreatic, cervical, hepatocellular, and esophageal squamous cell carcinoma [17–20]. However, the process of EMT-induced invadopodia in pancreatic cancer has not been elucidated. Hence, N- WASP could be a potential therapeutic target, with respect to cancer progression control.

Recently, we reported that lysyl oxidase-like 2 (LOXL2) is important for regulating EMT in pancreatic cancer [21]. However, the exact mechanism underlying LOXL2-mediated distant metastasis in pancreatic cancer has not been fully characterized. Therefore, in this present study, we investigated whether N-WASP expression is associated with LOXL2 by examining the development of pancreatic cancer using *in vitro* and *in vivo* models. We found that N-WASP mediates cancer progression by linking LOXL2 signaling to FAK signaling. Our findings indicate that LOXL2-induced N-WASP expression is a key regulator for tumor metastasis using invadopodia. These findings may facilitate the development of therapeutic strategies against pancreatic cancer.

## Materials and Methods

### Patients

From June 2002 to December 2012, 81 patients underwent radical curative resection for pancreatic cancer at Gangnam Severance Hospital, Yonsei University College of Medicine, Seoul, Korea. Among these patients, three were excluded, owing to poorly preserved tissue samples, incomplete clinicopathologic data, or loss to follow-up. And stage IV patients at diagnosis were excluded in this study. Hence, 81 patients were retrospectively reviewed. Patients were followed every 3 months during the first 12 months and then every 6 months after the first year. The study protocol was approved by the Institutional Review Board at Gangnam Severance Hospital, Yonsei University of Korea (3-2014-0153) and Institutional Animal Care and Use Committee (2019-0104) and complied with the Declaration of Helsinki. Informed consent was obtained from all participants.

### Cell culture

Mia PaCa-1, PANC-1, BXPC3, and AsPC-1 cells were obtained from American Type Culture Collection (ATCC) and cultured according to the ATCC guidelines. Cells were cultured in DMEM or RPMI (Biowest) supplemented with 10% fetal bovine serum (Biowest) and 1% antibiotic-anti mycotic (Gibco). Cells were incubated in a humidified atmosphere with 5% CO_2_ at 37°C.

### siRNA interference

For gene knockdown, the following small interfering RNA (siRNA) were purchased from Bioneer (Daejeon, KR): siLOXL2 (Bioneer, Cat. No 4017-1, sense: 5′- AGAUUCCGGAAAGCGUACA-3′, antisense: 5′-UGUACGCUUUCCGGAAUCU-3′) and si N-WASP (Bioneer, Cat.No 8976-1, sense: 5′- GUGCAUUAAUGGAAGUGAU-3′, antisense: 5′- AUCACUUCCAUUAAUGCAC-3′). Transfection was performed using Lipofectamine RNAiMAX (Invitrogen) according to the manufacturer’s instructions. Cells were harvested and processed 48–72 h post-transfection.

### Antibodies and western blot

To prepare the lysates, frozen pancreatic cancer tissues and cells were washed with ice-cold phosphate buffer solution (PBS) and lysed in RIPA lysis buffer. Proteins were separated via SDS-PAGE, and the protein bands were transferred to nitrocellulose membranes; after blocking the membranes with 5% skimmed milk, the membranes were incubated with primary antibodies (1:1000 dilution) against N-WASP (Abcam), phospho-N-WASP (Abcam), LOXL2 (Abcam), Vimentin (Cell Signaling), Snail (Cell Signaling), N-cadherin (BD Bioscience), E-cadherin (BD Bioscience), β-actin (Santa Cruz Biotechnology), or γ-tubulin (Sigma-Aldrich). The membranes were washed twice with Tris-buffered saline and Tween 20 (TBST) and then incubated with HRP-conjugated secondary antibodies (1:7,000 dilution, Cell Signaling Technology) in TBST with 3% skimmed milk. Bound antibodies were probed using the ECL solution (Bio-Rad). Chemiluminescent signals were captured using X-ray films (AGFA). All experiments were performed in triplicates.

### RNA isolation and qPCR

Cells and tissues were collected, and RNA was isolated using TRIZOL Reagent® (Sigma-Aldrich) according to the manufacturer’s instructions. Of the total isolated RNA, 0.2 µg was analyzed via reverse transcriptase PCR using one-step RT-PCR kit (Intron, KR). The first-strand cDNA was synthesized with 1 µg of RNA as template, using the RT-qPCR cDNA Synthesis Kit (Intron, KR), according to manufacturer’s instructions. RT-qPCR was performed using SYBR qPCR reaction mix (Applied Biosystems). Primer sequences used in this study are listed in Suppl. Table S1. The results are presented as relative mRNA expression level, calculated with the 2^−ΔΔCT^ method using Gapdh as reference gene.

### Migration and invasion assays

For scratch wound migration assay, 1.2−1.4 × 104 transfected cells were plated on a 24-well plate. Wound scratches were made 24 h after plating. Images of migrated cells were captured every 24 h using a microscope. For invasion assays, 8-µm pore size wells in a Transwell system (Corning Inc., New York, USA) were coated with Matrigel (1:50, Corning) for 1 h at room temperature. Transfected cells (2 × 104) were seeded on the apical side of the Transwell chamber (24-well insert) in serum-free media; growth media was added to the basal compartment. The cells were allowed to invade for 24 h. The remaining cells on the top of the chamber were gently scraped off using wetted cotton swabs. The cells that had invaded the basal side were fixed in methanol for 10 min, stained with 0.2% crystal violet, and washed multiple times with 3’ DW. Migration and invasion assays were performed in triplicate and repeated three times independently.

### Immunocytochemistry

Transfected cells were fixed with 3.7% paraformaldehyde for 10 min and permeabilized with 0.1% Triton X-100 for 30 min. Cells were washed with PBS thrice and incubated with 1% bovine serum albumin for 1 h at room temperature. Cells were incubated with primary antibodies (LOXL2, N-WASP, ARP2, phalloidin iFlour-488; Abcam) overnight at 4°C (1:100 to 1:1000) and incubated at room temperature with Alexa Fluor 488 or 555 conjugated secondary antibodies (1:1000). FluoroShield mounting medium with DAPI (Abcam) was used to stain and mount cells for 5 min. Immunostained cells were observed under a Carl Zeiss LSM780 confocal microscope.

### Invadopodia formation assay

FITC-gelatin invadopodia assays were performed as per the manufacturer’s procedure (QCM™ Gelatin Invadopodia Assay, Millipore, St. Louis, MO, USA). Briefly, 8-well chamber slides were coated with poly-L-lysine for 20 min at room temperature, washed with PBS, fixed with glutaraldehyde for 15 min, and washed with DPBS thrice. After washing, a gelatin mixture (1:5, fluorescein gelatin:unlabeled gelatin) was applied to each well and incubated was carried out at room temperature for 10 min. Substrates disinfected with 70% ethanol in sterile water were incubated at room temperature for 30 min and protected from light. To eliminate residual free aldehydes, growth medium was added to each well, and the wells were incubated at room temperature for 30 min with protection from light. Cells were plated in wells at 20%–80% confluence and incubated for 24 h. After incubation, slides were fixed with 3.7% formaldehyde in DPBS for 30 min at room temperature. Treatment with blocking or permeabilization buffer for fluorescent staining was performed for 1 h at room temperature. Slides were incubated in TRITC-phalloidin and DAPI in fluorescent staining buffer for 1 h. The chamber was removed, and the slide was covered with mounting media. Imaging of invadopodia and ECM degradation was performed using fluorescence confocal microscopy (Carl Zeiss 780).

### Knockdown in stable cell lines

Five different short hairpin ribonucleic acid (shRNA) N-WASP sequences in a lentiviral vector (pLKO.1-Puro) and controlled shRNA were purchased from Sigma-Aldrich. For the production of lentiviruses and transfection to HEK293 cells, Lipofectamine 3000 (Invitrogen) was used. PANC-1 cells were infected with the collected and purified viral particles; cells were stably selected and maintained with 2 μg/ml puromycin treatment. The shRNA sequence that sufficiently knocked down N-WASP expression was GCACAACTTAAAGACAGAGAA (TRCN0000123061, Sigma-Aldrich).

### Immunoprecipitation

To analyze protein binding, cell lysates were incubated with anti-FAK or anti-ARP2 antibodies overnight at 4°C, and immune complexes were pulled down with protein A/G magnetic beads (EMD Millipore, USA). The interacted proteins were eluted in a denaturing SDS sample buffer, loaded into wells for SDS-PAGE, and following resolution via SDS- PAGE, transferred to a membrane. The membrane was blocked, then incubated with indicated primary antibodies, and then stained using the ECL solution.

### Pancreatic cancer orthotropic model

All animal studies were conducted with an approved protocol proposal from the animal ethics committee of Yonsei University College of Medicine (approval #2019-0104). BALB/c nude mice (6 weeks old, female) were purchased from Orient Bio. The pancreas was surgically exposed through an abdominal excision under anesthesia with an intraperitoneal injection (i.p.) of Alfaxan (25 mg/kg). Human pancreatic cancer cells were inoculated directly into the pancreas of mice using a 30 G needle (BD Biosciences). PANC-1 cells (1 × 106) were mixed with serum-free DMEM and Matrigel (1:1) and injected at a volume of 50 μl. To prevent cancer cells from leaking, the excision was closed after covering the injection site with the surge cell. After the operation, mice were warmed and monitored until conscious, following which they were placed in HEPA-filtered cages with feed and water. After 12 weeks, mice were sacrificed and examined for tumor spreading through macroscopic and microscopic observations using hematoxylin and eosin (H & E) staining. The pancreas, spleen, liver, and lung were examined using H & E-stained sections.

### Immunohistochemical (IHC) staining

Serial sections (5 µm) of each block were adhered to poly-L-lysine- covered slides and incubated at 62°C for 60 min. After deparaffinization and rehydration, the sections were heated in citrate buffer (10 mM, pH 6.0) for 15 min and stained with an antibody. Normal pancreas tissues within the block were used as positive controls. IHC staining was categorized as negative, “1+”, “2+”, or “3+” in high-power fields (200×) according to the staining intensity. High expression was assigned for the scores “2+” and “3+”. The staining intensity was assessed by two pathologists, who were blinded to the clinical outcomes.

### Statistical methods

For the statistical analysis, unpaired *t*-test and ANOVA were performed. Differences were considered statistically significant at *p < 0.05; **p ≤ 0.01. The tests were performed using GraphPad Prism version 8.01 (GraphPad Software).

## Results

### N-WASP is highly expressed in pancreatic cancer cell lines and tissues

**Figure 1:**
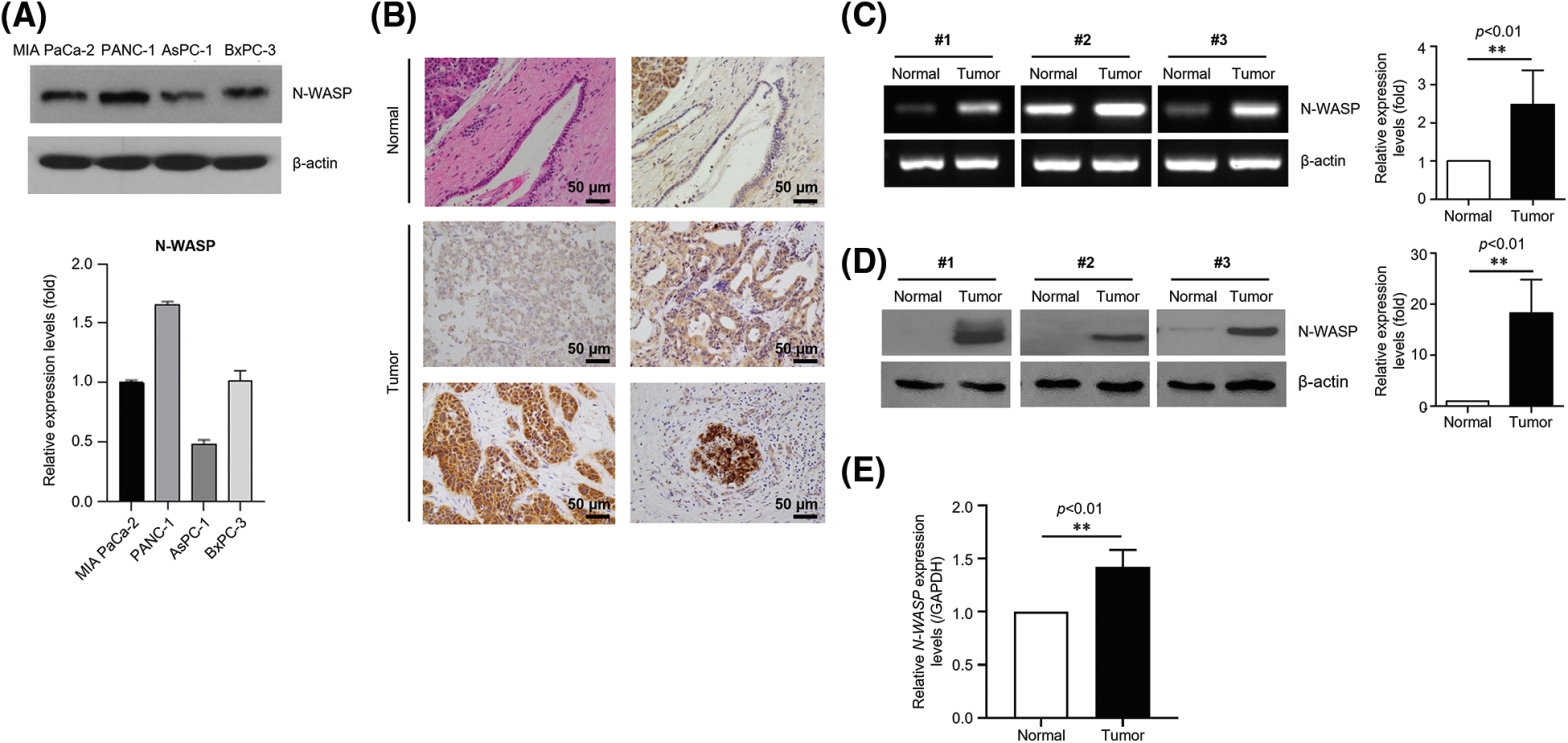
N-WASP is highly expressed in pancreatic cancer cell lines and tissues. (A) Relative mRNA expression of N-WASP in pancreatic cancer cell lines. (B) Immunohistochemical staining of N-WASP in human pancreas tissues (upper, normal pancreas; lower, pancreatic cancer). (C) Relative mRNA expression levels of N-WASP in human pancreatic cancer tissues (N, normal tissue; T, tumor). (D) N-WASP protein expression in human pancreatic cancer tissues, as per western blot analysis. (E) Real-time PCR results of N-WASP expression in human pancreatic cancer tissues. All data are presented mean ± SD. **p < 0.01. All experiments were performed at least three times.

To investigate the effects of N-WASP and its underlying molecular mechanisms, N-WASP expression was analyzed in a series of pancreatic cancer cell lines (MIA PaCa-2, PANC-1, AsPC-1, and BxPC-3). N-WASP was detected in all pancreatic cell lines (Fig. 1A). We investigated N-WASP expression in PDAC samples from 81 patients. In PDAC tissue, N-WASP overexpression was observed, whereas normal pancreatic tissue exhibited low expression of N-WASP, as assessed via IHC staining, western blotting, and RT-qPCR (Figs. 1B–1E).

### Clinical characteristics and patterns of recurrence according to N-WASP status

Patient characteristics, including expression of N-WASP, were evaluated using IHC staining (described in Table 1). There was no significant difference in T stage, N stage, tumor stage, cell differentiation, perineural invasion, and lymphovascular invasion among patient groups, based on N-WASP expression levels. N-WASP-high group showed a pattern of notably more distant metastasis (*p* = 0.004), compared to the N-WASP-low group (Table 2).

**Table 1:** Clinical characteristics of patients according to N-WASP expression

	N-WASP-low (n = 56)	N-WASP-high (n = 25)	*p* value
Age	63.66 ± 10.15	64.24 ± 9.56	0.925
Sex			
Men	26 (46.4%)	14 (56%)	0.426
Women	30 (53.6%)	11 (44%)	
T stage (8th)			
T1	9 (16.1%)	4 (16.0%)	0.414
T2	36 (64.3%)	17 (68%)	
T3	11 (19.6%)	3 (12%)	
Unknown	0 (0%)	1 (4%)	
N stage (8th)			
N0	19 (33.9%)	6 (24%)	0.645
N1	28 (50%)	15 (60%)	
N2	9 (16.1%)	4 (16%)	
Stage (8th)			
IA	5 (9.1%)	1 (4%)	0.561
IB	8 (14.5%)	2 (8%)	
IIA	6 (10.9%)	2 (8%)	
IIB	27 (49.1%)	15 (60%)	
III	9 (16.4%)	4 (16%)	
Unknown	0 (0%)	1 (4%)	
Perineural invasion			
Positive	39 (69.6%)	20 (80%)	0.622
Negative	14 (25%)	4 (16%)	
Unknown	3 (5.4%)	1 (4%)	
Lymphovascular invasion			
Positive	22 (39.3%)	15 (60%)	0.223
Negative	31 (55.4%)	9 (36%)	
Unknown	3 (5.4%)	1 (4%)	
Cell differentiation			
Well differentiation	11 (19.6%)	2 (8%)	0.168
Moderate differentiation	39 (69.6%)	16 (64%)	
Poorly differentiation	5 (8.9%)	5 (20%)	
Undifferentiation	1 (1.8%)	2 (8%)	

**Table 2:** Comparison of recurrence pattern among N-WASP-low and N-WASP-high patients

	N-WASP-low (n = 46)	N-WASP-high (n = 17)	*p* value
Local metastasis	27 (58.7%)	3 (17.6%)	0.004
Distant metastasis	19 (41.3%)	14 (82.4%)	

### N-WASP knockdown inhibits EMT in PDAC and reduces motility and invasiveness in pancreatic cancer cells

**Figure 2:**
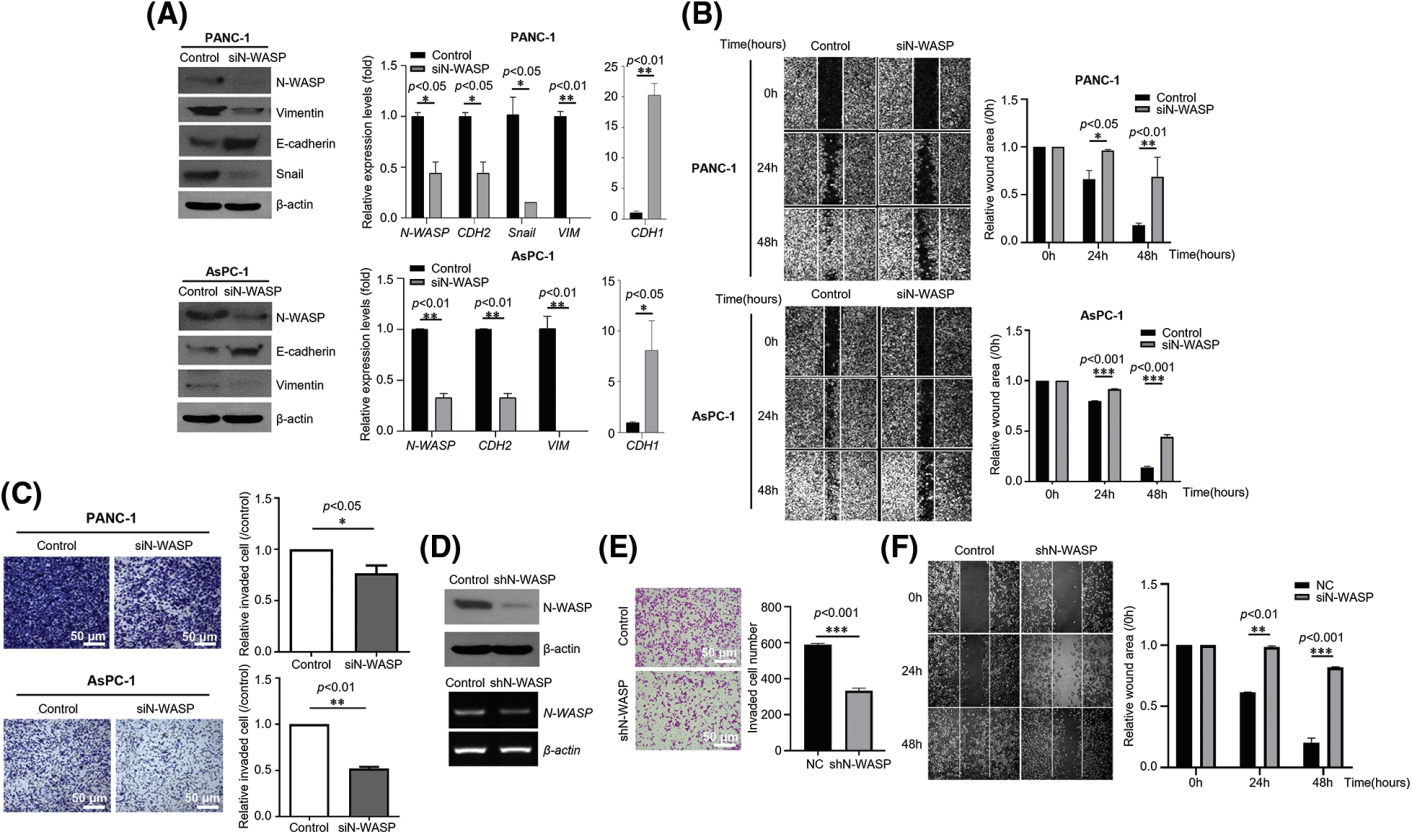
Effects of N-WASP inhibition in pancreatic cancer cells on EMT and cell migration. (A) PANC-1 and AsPC-1 cells were transfected with negative control (NC) and N-WASP siRNA. Western blot analysis was performed for EMT markers. (B) Cell monolayers transfected with negative control or N-WASP siRNA were scraped with a razor blade and then incubated in a fresh medium containing 5% FBS. After incubation for the indicated time points, the number of cells that migrated across the wound was counted. Representative photographs and wound area are presented as mean ± SD. (C) Transwell migration and invasion assays of PANC-1 and AsPC-1 cells transfected with negative control or si-N-WASP. (D) Stable knockdown of N-WASP protein and mRNA in PANC-1, as detected via western blot analysis (Upper), RT-PCR (Lower), (E) Transwell invasion assay, and (F) Wound healing migration assay. All experiments were performed at least three times. *p < 0.05; **p < 0.01; ***p < 0.001.

To evaluate whether N-WASP expression affects pancreatic cancer cells, N-WASP knockdown was performed in PANC-1 and AsPC-1 cells using siRNA. As shown in Fig. 2A, N-WASP-knockdown cells exhibited a significant increase in E-cadherin expression levels and a significant reduction in Snail and Vimentin expression levels. Moreover, wound healing assay results revealed that the wound width was significantly reduced in N-WASP-knockdown cells (Figs. 2B and 2F). The results indicated that N-WASP knockdown suppressed cell migration. Furthermore, Transwell assay was performed to determine whether N-WASP could affect pancreatic cancer cell migration and invasion. The results revealed an approximately 70% reduction in cell migration and 50% reduction in cell invasion in N-WASP siRNA-transfected PANC-1 and AsPC-1 cells (Figs. 2C and 2E). These results suggest that inhibition of N-WASP expression suppresses PDAC progression.

### Identification of potential target genes of N-WASP in pancreatic cancer

**Figure 3:**
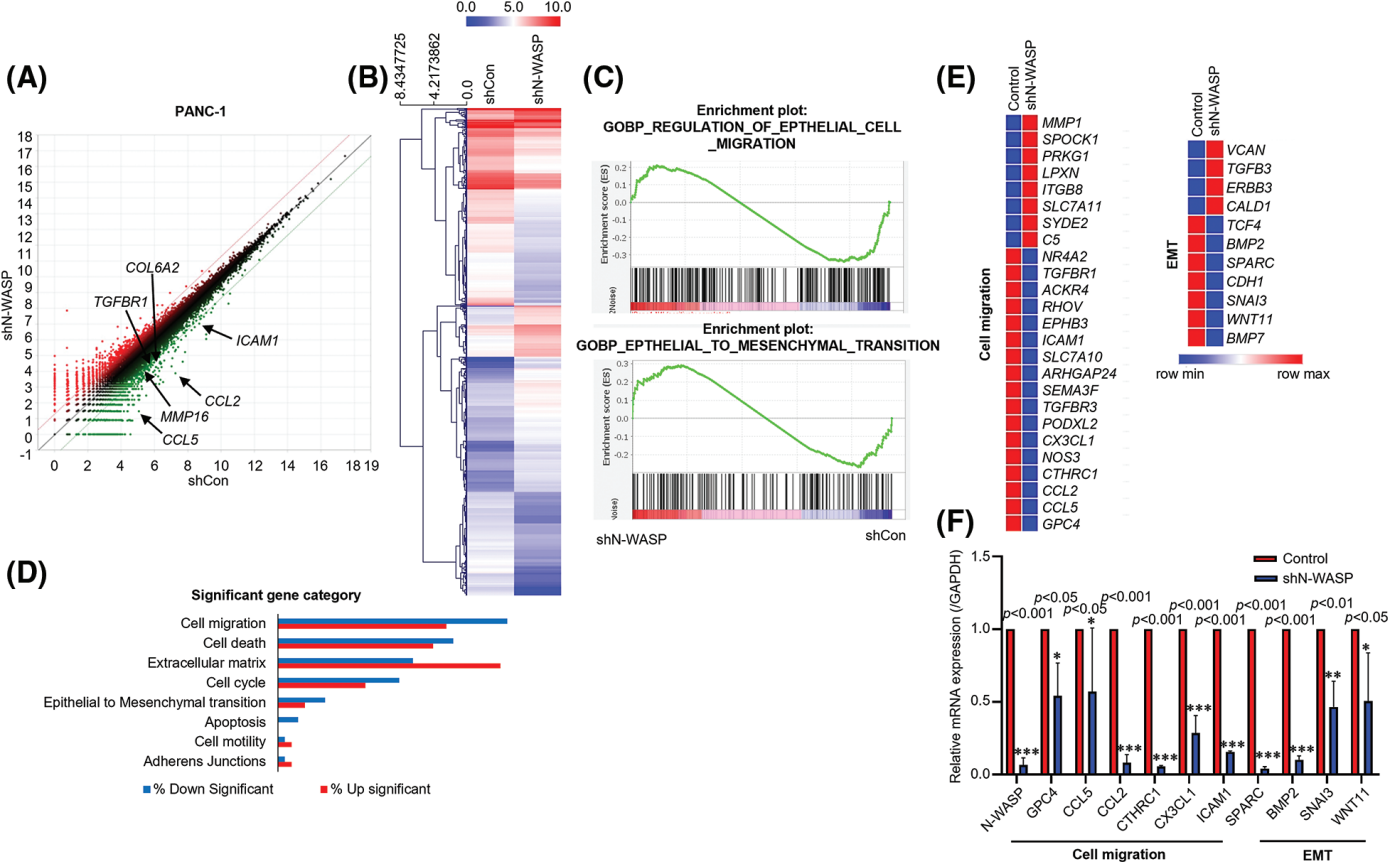
N-WASP expression is positively correlated with PDAC development. (A and B) Heat map showing the relative expression of N-WASP and genes positively correlated with N-WASP from RNA sequencing data (red, high expression; blue, low expression). (C) The gene set enrichment analysis (GSEA) plot indicated that high expression of genes involved in EMT was significantly positively correlated in PANC-1 cells. (D) Functional annotations of significantly downregulated and upregulated genes (with fold change >2 and *p* < 0.05) were performed in DAVID web-based tool. Bars show the fold enrichment of pathways that were significantly downregulated or upregulated in N-WASP from advanced stages *vs*. early stages. (E) Heat maps for selected pathways show the Z-score for genes that were differentially expressed. (F) Expression of the genes involved in cell migration and EMT quantified, as per qPCR analysis. *p < 0.05; **p < 0.01; ***p < 0.001. All experiments were performed at least three times.

To investigate how N-WASP promotes PDAC progression, we performed RNA sequencing (RNA-seq) to evaluate changes in target gene expression, triggered by N-WASP knockdown in PANC-1 cells. using RNA-seq results, genes showing changes in expression were identified after N-WASP knockdown. Additionally, several genes showed more than a two-fold difference in expression levels, compared to shControl cells (Figs. 3A and 3B). We further confirmed the potential pathway via gene set enrichment analysis (GSEA) pre-ranked tool analysis, performed on all genes. GSEA revealed that N-WASP enrichment was associated with genes in “HALLMARK EMT” and “HALLMARK Cell migration” (Fig. 3C). Then, gene ontology (GO) analysis revealed an association of the differentially expressed genes with cell migration and EMT (Figs. 3D and 3E). Among the genes showing lower expression in shN-WASP-transfected cells, there were many genes known to be associated with EMT and cell migration (e.g., GPC4, CCL5, CCL2, CTHRC1, CX3CL1, ICAM1, SPARC, BMP2, SNAI3, and WNT11). The expression levels of some of these genes were verified using qRT-PCR after N-WASP knockdown in PANC-1 cells (Fig. 3F).

### FAK-dependent phosphorylation of N-WASP is regulated by LOXL2

**Figure 4:**
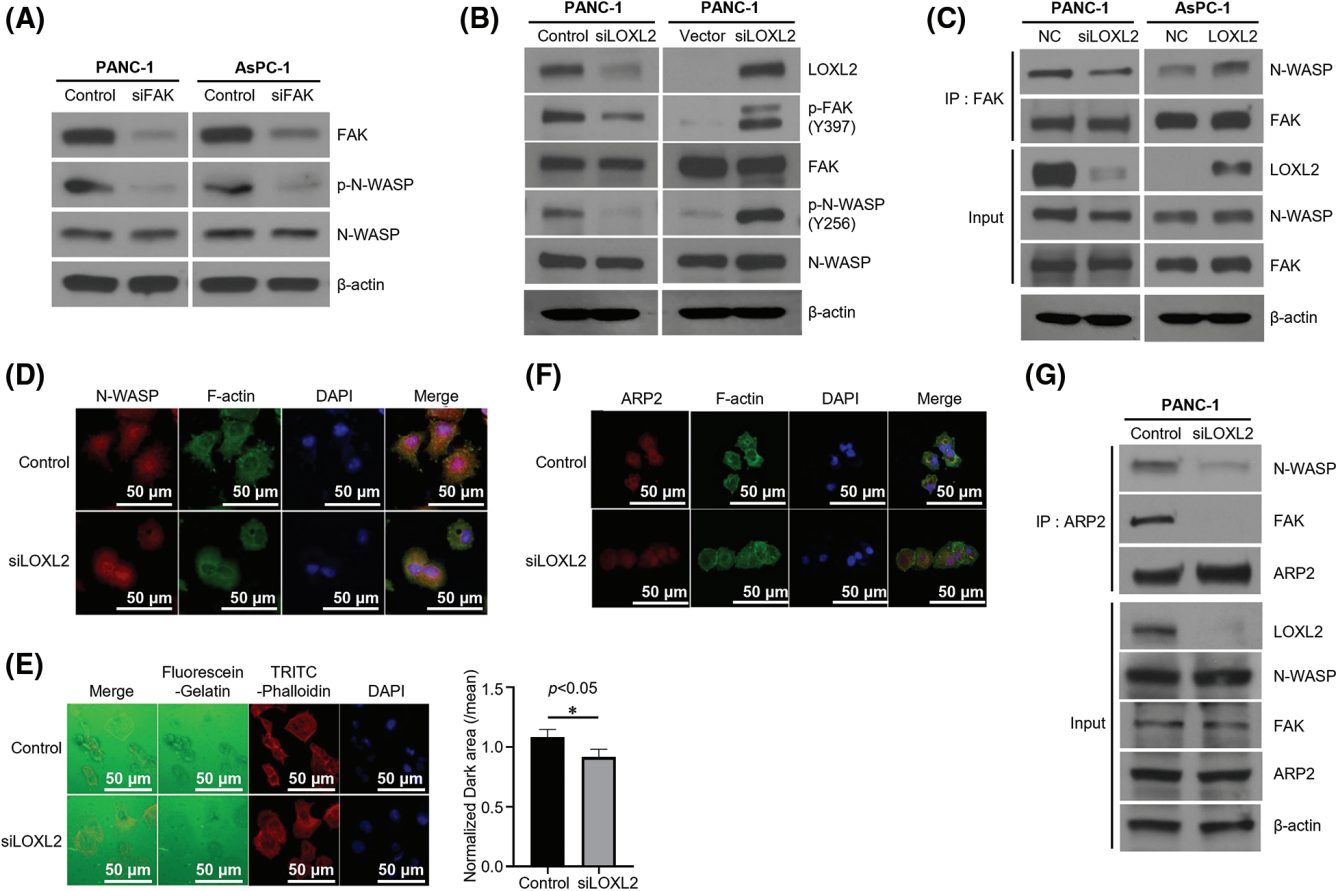
FAK-dependent phosphorylation of N-WASP is regulated by LOXL2. (A) PANC-1 and AsPC-1 cells were transfected with negative control and FAK siRNA. Western blot analysis was performed to assess EMT markers. (B) Cells transfected with LOXL2 siRNA or pcDNA-LOXL2 vector. Western blot analysis using indicated antibodies. γ-tubulin was used as the loading control. (C) Immunoprecipitation (Ip) of endogenous FAK and N-WASP. Lysates from siLOXL2-transfected PANC-1 cells were immunoprecipitated with anti-FAK antibody and western blotting was performed using indicated antibodies. (D) Immunofluorescence staining of N-WASP (red) affected by LOXL2 silencing. The F-actin cytoskeleton was stained with green fluorescence and nuclei were stained with DAPI (blue). (E) Invadopodia formation assay. Dark areas of gelatinase activity (black) reveal invadopodia-mediated degradation. Nuclei are labeled with DAPI (blue), and F-actin cytoskeleton is labeled with TRITC-Phalloidin (red). Matrix degradation was quantified (right) by measuring the dark area of five individual fields; results are normalized and presented as mean ± SD (Unpaired *t*-test, *p* = 0.01). *p < 0.05. (F) Immunoprecipitation of endogenous ARP2 with N-WASP and FAK using LOXL2 siRNA-transfected cell lysate. (G) Validation of translocation of ARP2 via LOXL2 silencing. Immunofluorescence staining for endogenous ARP2 (red) and the F-actin cytoskeleton (green) is shown. All experiments were performed at least three times.

Previous studies revealed that the FAK/LOXL2 axis is crucial for regulation of EMT and metastasis in PDAC. To determine whether LOXL2 affects N-WASP, we evaluated the correlation between FAK-dependent phosphorylation of N-WASP and LOXL2 expression. FAK-knockdown cells exhibited a significant reduction in the expression of phosphorylated N-WASP (p-N-WASP) (Fig. 4A). LOXL2- knockdown PANC-1 cells exhibited a significant reduction in the expression of phosphorylated FAK (p-FAK) and p-N-WASP. We also analyzed AsPC-1 cells in which LOXL2 was overexpressed. LOXL2- overexpressing PANC-1 cells showed an increase in the expression of p-FAK and p-N-WASP (Fig. 4B). As shown in Fig. 4C, LOXL2 knockdown decreased the efficiency of the interaction between N-WASP and FAK. These results suggest that the FAK/N-WASP axis is significantly associated with LOXL2 expression in pancreatic cancer cells.

### LOXL2 influences actin filament regulation and invadopodia formation ability of N-WASP

Invadopodia formation is essential for invasion and metastasis. Cortactin was noted to play a critical role in invadopodia formation and promotion of cell motility and invasion (Figs. 4D and 4E). Furthermore, LOXL2 knockdown decreased the efficiency of the interaction between N-WASP and ARP2 (Figs. 4F and 4G). These results suggest that inhibition of the N-WASP/FAK/LOXL2 axis could reduce the progression of PDAC. To determine the effects of LOXL2 on invadopodia formation and investigate the expression of F-actin in LOXL2-knockdown PANC-1 cells, changes in cell morphology and invadopodia were recorded via immunofluorescence staining.

### Regulation of N-WASP-LOXL2 axis affects invasiveness and expression of EMT markers in pancreatic cancer cells

**Figure 5:**
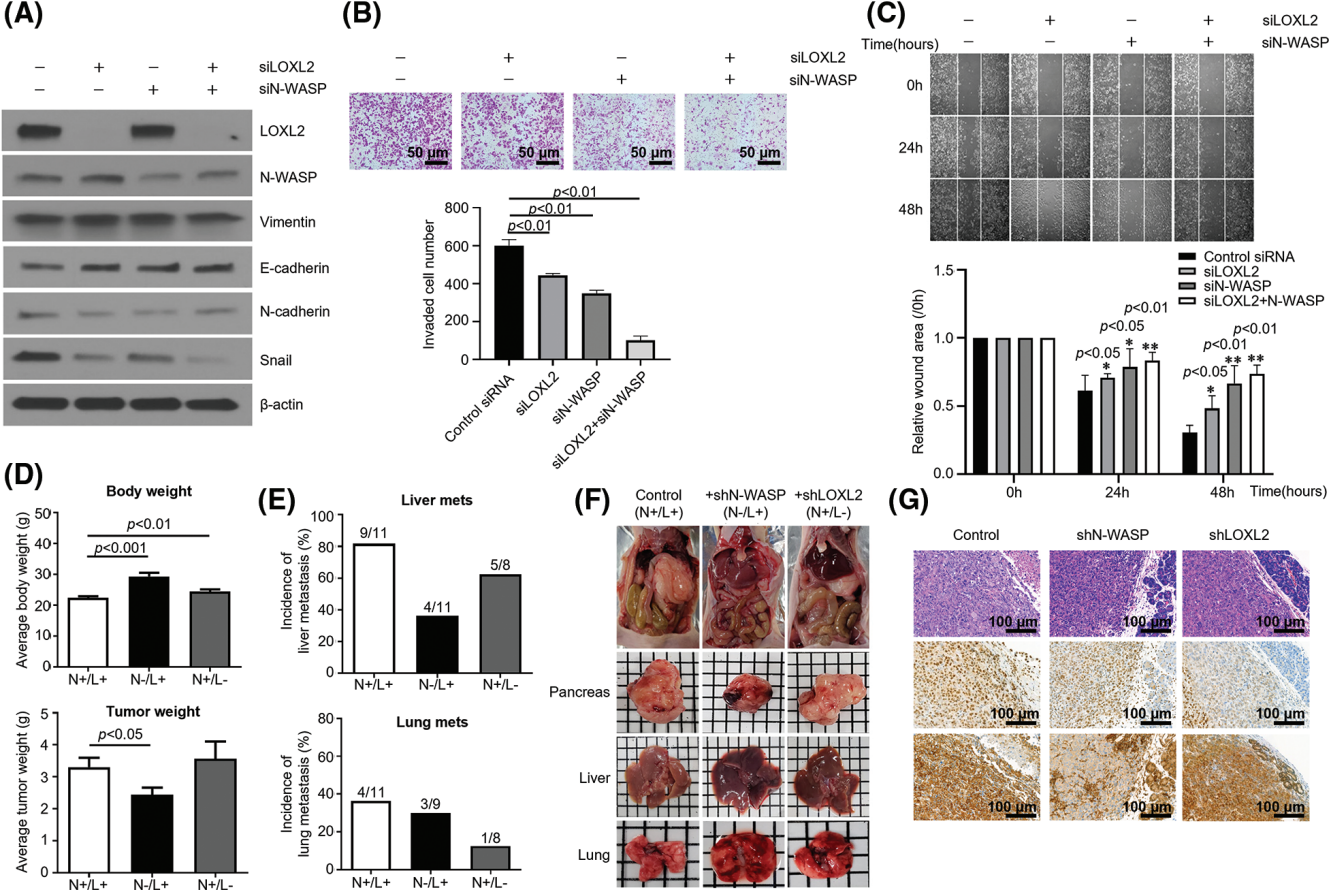
Inhibition of N-WASP reduces metastasis of pancreatic cancer to the liver and lungs. PANC-1 cells were transfected with LOXL2 and N-WASP siRNA. (A) Western blot analysis was performed to assess EMT markers. (B) Transwell invasion assay and (C) wound healing migration assay. (D) Average body weight and tumor weight of orthotopic pancreatic cancer mouse model. Control shRNA (N+/L+)-, N-WASP shRNA-, or LOXL2 shRNA-transfected PANC-1 cells (1 × 106) were orthotopically injected into the pancreas of nude mice (n = 5 per group). Tumor weight and body weight were measured after the mice were euthanized at 12 weeks. (E) Percentage of mice with lung and liver metastases in each group. (F) A representative image of the tumor mass and orthotopic mouse from each group. (G) Immunohistochemical staining of N-WASP and LOXL2 in mouse pancreas tissues. *p < 0.05; **p < 0.01; ***p < 0.001. All experiments were performed at least three times.

To further explore the detailed molecular mechanisms by which the N-WASP-LOXL2 axis inhibits EMT, we used siRNAs to knockdown N-WASP and LOXL2. Western blot analysis was used to compare groups having knockdown of either or both of N-WASP and LOXL2. The group with combined knockdown of N-WASP and LOXL2 showed a significant reduction in levels of EMT markers, in comparison with groups wherein either N-WASP or LOXL2 was knocked down (see N-cadherin, Vimentin, and Snail levels in Fig. 5A). Silencing of N-WASP and LOXL2 significantly repressed migration and invasion of PANC-1 cells (Fig. 5B). In concordance with these findings, wound healing test revealed that combined knockdown of N-WASP and LOXL2 inhibits the wound healing ability (Fig. 5C). These data suggest that the N-WASP-LOXL2 axis promotes migration, invasion, and morphology change in pancreatic cancer cells.

### Inhibition of N-wasp reduces metastasis of pancreatic cancer to the liver and lungs

To confirm whether LOXL2-N-WASP impacts tumorigenesis of pancreatic cancer *in vivo*, PANC-1 cells transfected with shN-WASP or control vector were injected into the pancreas of nude mice. On day 84 after the orthotopic modeling, the body weight of the shN-WASP group was remarkably higher than of the PANC-1 group. Similarly, the average tumor weight was lower in the shN-WASP group (Fig. 5D). Both liver and lung metastasis occurred to a lower degree in the shN-WASP group, compared to the PANC-1 group. N-WASP silencing can repress the pancreatic cancer progression *in vivo*, suggesting that N-WASP performs a crucial function in pancreatic cancer metastasis and proliferation (Figs. 5D and 5E). With respect to microscopic features of the primary tumor in an orthotopic model of PANC-1 group, cancer cells expressed LOXL2 and N-WASP, as confirmed via IHC staining for each primary antibody (Fig. 5G). LOXL2 expression levels were similar in the shN-WASP and PANC-1 groups, whereas N-WASP expression levels decreased in the shN-WASP group (Fig. 5G). LOXL2 expression levels decreased and those of N-WASP were similar or slightly lower in the shLOXL2 group (Fig. 5G; H & E, x200).

## Discussion

We demonstrated, via an integrated *in vitro*, *in vivo*, and clinical investigation, that signaling through the LOXL2-N-WASP axis contributes to tumor invasion and metastasis in pancreatic cancer. Our results showed that N-WASP is overexpressed in both pancreatic cancer lines and human pancreatic cancer tissues. Moreover, high expression of N-WASP was associated with increased distant metastasis and poor prognosis *in vivo* and clinically.

Among the five members of the WASP family, namely, WAVE-1–3, N-WASP, and WASP, N-WASP and WAVE-1 are crucial for invadopodia formation and ECM degradation, respectively. Thus, distinct pathways activate branched actin assembly for invadopodia formation [22–25]. Invadopodia are subcellular structures that are selectively invasive, as compared to subcellular structures of non-invasive cancer cells, and are involved in a pivotal process in cancer invasion [5,10,26].

We showed that N-WASP promotes proliferation and migration in pancreatic cancer cells. N-WASP increased colony formation *in vitro* and distant metastasis *in vivo*. The knockdown of N-WASP inhibited both cell proliferation and migration. Additionally, high N-WASP expression level was correlated with a high distant metastasis potential. The results of experiments on mice revealed N-WASP to be essential for invasion and metastasis in pancreatic cancer. We further investigated the correlation of N-WASP expression with clinicopathologic features via IHC analysis of 81 pancreatic cancer tissues. N-WASP expression correlated with distant metastasis after surgery.

Our previous study showed that LOXL2 expression levels were higher in human pancreatic cancer cells than in cells of normal pancreas. Additionally, higher LOXL2 expression levels in pancreatic cancer were associated with a significantly greater potential for distant metastasis [21].

Invadopodia and podosomes share many common functional and molecular characteristics, including dependence on Src kinase and branched actin assembly, as well as common molecular components, including focal adhesion proteins, integrins, and proteases. Many molecules that localize to focal adhesion proteins are found in invadopodia or podosomes, including cytoskeletal proteins (e.g., alpha-actinin, VASP, and zyxin), integrin linkers (e.g., vinculin and talin), and signaling molecules (e.g., FAK, paxillin, p130Cas, and ERK) [27–30].

Moreover, positive modulation of the FAK and SRC pathways by LOXL2 has been reported, which contributes to cell migratory behavior. Recently, Baker et al. showed that LOXL2 activated fibroblasts through integrin-mediated FAK activation in tumor cell invasion and metastasis [31]. LOXL2 plays an integral role in EMT promotion and invasiveness of pancreatic cancer cells. Our *in vitro* study indicates that LOXL2 activates EMT- like processes in pancreatic cell lines that are associated with invasive and migratory properties. Moreover, LOXL2 promotes the activation of FAK/SRC and is involved in regulation of expression of CDH1, Snail, and L1CAM, all of which are related to EMT and invasiveness of pancreatic tumor cells.

Therefore, we analyzed the signaling associated with the LOXL2-FAK-N-WASP axis. Specifically, to verify the effect of N-WASP on distant metastasis, an orthotopic mouse model was constructed, which showed significant results. Accordingly, it is important to determine the role and mechanism of N-WASP in pancreatic cancer, specifically its role in metastasis. We elucidated the signaling mechanism of N-WASP using RNA transcriptome sequencing and analysis of related genes.

In conclusion, we showed that N-WASP plays a crucial role in regulating expression of genes associated with cell migration and EMT. Our results suggest that N-WASP function affects the metastasis of cancer cells. This study proved a correlation between N-WASP expression in pancreatic cancer and distant metastasis. The findings support emerging evidence regarding the importance of the LOXL2-N-WASP axis in regulation of metastasis of pancreatic cancer.

## Supplementary Materials



## Data Availability

The datasets generated and/or analysed during the current study are available from the corresponding author on reasonable request.
