# Supplementary material for: Neural Wiskott-Aldrich syndrome protein (N-WASP) promotes distant metastasis in pancreatic ductal adenocarcinoma via activation of LOXL2
Source: Oncol Res. 2024 Mar 20;32(4):615–24. doi: 10.32604/or.2024.044029 (PMC10972719; doi:10.32604/or.2024.044029)
Supplement: Supplementary file 1 [file OncolRes-32-44029-s001.docx]

**Supplementary Table 1. qPCR primer sequence**

| Gene | Direction | Sequence |
| --- | --- | --- |
| LOXL2 | Forward | TGCCTGCAAGCACACGGAGGAT |
|  | Reverse | TCCGAATGTCCTCCACCTGGAT |
| N-WASP | Forward | AAAGTGGAGCAGAACAGTCGGC |
|  | Reverse | GTTGGTGGTGTAGACTCTTGGC |
| GPC4 | Forward | GGAGATGTCGTGAGCAAGGTCT |
|  | Reverse | CATGGCTTCACAGTCACGAGAC |
| CCL5 | Forward | GCTGCTTTGCCTACATTGCCCG |
|  | Reverse | ACACACTTGGCGGTTCTTTCGG |
| CCL2 | Forward | AGAATCACCAGCAGCAAGTGTCC |
|  | Reverse | TCCTGAACCCACTTCTGCTTGG |
| CTHRC1 | Forward | CAGGACCTCTTCCCATTGAAGC |
|  | Reverse | GCAACATCCACTAATCCAGCACC |
| CX3CL1 | Forward | ACAGCACCACGGTGTGACGAAA |
|  | Reverse | AACAGCCTGTGCTGTCTCGTCT |
| ICAM1 | Forward | AGCGGCTGACGTGTGCAGTAAT |
|  | Reverse | TCTGAGACCTCTGGCTTCGTCA |
| SPARC | Forward | CTTCGGTTTCCTCTGCACCATC |
|  | Reverse | TGAGACACCTCTCCAGTTGCTG |
| BMP2 | Forward | TCAAGCCAAACACAAACAGC |
|  | Reverse | AGCCACAATCCAGTCATTCC |
| SNAI3 | Forward | ATCAATGGTGCCTGCTCTGC |
|  | Reverse | CAGAGCTTCCTCGATCCGTG |
| WNT11 | Forward | CTGTGAAGGACTCGGAACTCGT |
|  | Reverse | AGCTGTCGCTTCCGTTGGATGT |
| GAPDH | Forward | GTCTCCTCTGACTTCAACAGCG |
|  | Reverse | ACCACCCTGTTGCTGTAGCCAA |
| β-actin | Forward | AGAGCTACGAGCTGCCTGAC |
|  | Reverse | AGCACTGTGTTGCGTACAG |
